# “I’m not alone”: a qualitative report of experiences among parents of children with eating disorders attending virtual parent-led peer support groups

**DOI:** 10.1186/s40337-022-00719-2

**Published:** 2022-12-15

**Authors:** Laura Grennan, Maria Nicula, Danielle Pellegrini, Kelly Giuliani, Erica Crews, Cheryl Webb, Maria-Rosa Gouveia, Techiya Loewen, Jennifer Couturier

**Affiliations:** 1 McMaster University, 1280 Main St W, Hamilton, ON L8S 4L8 Canada; 2 Reach Out Centre for Kids, 471 Pearl St, Burlington, ON L7R 4M4 Canada; 3 McMaster Children’s Hospital, 1200 Main St W, Hamilton, ON L8N 3Z5 Canada; 4 Phoenix Wings Eating Disorder Recovery Initiative, 872 Devonshire Ave, Woodstock, ON N4S 5R8 Canada

**Keywords:** Eating disorders, Peer support, Parents, Caregivers, Children, Adolescents, Virtual

## Abstract

**Background:**

The treatment for children with eating disorders (EDs) requires extensive involvement of parents. The parents of children with EDs have voiced a need for greater support, including connecting with other parents with lived experience of caring for a child with an ED. We aimed to qualitatively explore parental experiences of these groups, including their benefits and areas for improvement.

**Methods:**

This study examined the delivery of four virtual parent-led peer support groups in Ontario, Canada for parents of children with EDs with approximately 10 parent participants per group and two parent facilitators leading each group. Parents (n = 44) were asked to attend 12 bi-weekly support group sessions over 6 months, and then complete an individual end-of-study qualitative interview. Interview data were analyzed using content analysis, following the qualitative description design.

**Results:**

Thirty-six parents completed the end-of-study qualitative interview. Participants shared their experiences and impressions related to the group’s structure and content. Notable helpful aspects of the group included being able to receive support from those with similar experiences, access to education and resources about EDs, and being able to support others. Suggestions for improvements were made, which included organizing groups according to the child’s ED diagnosis or duration of illness.

**Conclusion:**

The findings indicate that this intervention is acceptable to parents and is perceived as helpful. Future research is needed to strengthen this support group model and to study its effects for parents in different settings and for parents of children with various EDs.

*Trial registration*: ClinicalTrials.gov NCT04686864.

**Supplementary Information:**

The online version contains supplementary material available at 10.1186/s40337-022-00719-2.

## Plain English summary

The treatment of children who have eating disorders (EDs) requires extensive involvement from parents. Parents have voiced a need for greater support, and one way to meet this need is through peer support. Using end-of-study interviews, this study qualitatively described the experiences of parents of children who have EDs who participated in virtual parent-led peer support groups (vPLPSGs) for 6 months. Our findings suggest that vPLPSGs are beneficial for parents of children who have EDs. Participants had positive perceptions of the group organization and structure, the parent facilitators, and other parent attendees, and valued the support and educational resources received in the group. Parents noted it was particularly helpful to be able to speak with other parents who understood their experiences, which helped them feel less alone in their difficulties with their child. Parents also noted an improvement in their relationship with their child who has an ED, and believed that these groups should be readily available and accessible. Suggestions for improvement included dividing groups based on children’s ED diagnosis, duration of illness, and geographical location. Further expansion and study of these groups is warranted, as they appear to be a valuable support for families affected by EDs.

**Supplementary Information:**

The online version contains supplementary material available at 10.1186/s40337-022-00719-2.

## Background

Family-based treatment—the most widely supported and efficacious treatment for children and adolescents with eating disorders (EDs) [1, 2]—places substantial responsibility on parents and/or caregivers [3]. This includes deciding what and when their child eats, how much is eaten, the amount of physical activity their child engages in, as well as interrupting disordered eating behaviours [3]. Although highly effective, parents have reported struggling throughout the FBT process, expressing feelings of guilt and anxiety [4, 5]. These feelings have been exacerbated by the COVID-19 pandemic, as parents and caregivers juggled increased practical demands during lockdowns, changes in the delivery of treatment for their child with an ED, and stresses related to acquiring COVID-19 and following social distancing guidelines [6].

The benefits of parent-led peer support groups (PLPSGs) for parents of children with disabilities have been outlined in the literature [7–9]. Such groups involve regular meetings among parents of children with disabilities with the intention of providing support and sharing information with each other, led by an experienced parent who also has a child with a disability [7–9]. These groups have been found to alleviate parental burden and psychological distress by reducing social isolation, facilitating connections with others, sharing problem solving and coping skills, and establishing a sense of power and self-efficacy [7–9]. Parental self-efficacy is tremendously important in the ED field, as it has been associated with better outcomes and faster recovery for children with EDs [10, 11].

While there are reports of clinician-facilitated parent support groups [12] and internet chat support groups [13] in the pediatric ED field, no reports exist for PLPSGs specifically for parents of children and adolescents with EDs, with the exception of one recent report on a support group for parents of those with an ED with comorbid autism spectrum disorder (ASD) [14]. This is despite parents expressing a need for increased support at all stages of treatment [5, 15, 16]. In fact, parents of children and adolescents with EDs have specifically indicated a desire for peer support for themselves [17], and in some cases have created their own peer support groups to meet this perceived need [18].

In the *Canadian Practice Guidelines for the Treatment of Children and Adolescents* [1] and its addendum on virtual care recommendations [19], in-person and virtual peer support groups for parents and/or caregivers were highlighted as a gap in the ED care continuum that are acutely needed. Previous research also demonstrates that peer support is an important component of care within a full suite of ED services [20]. Given the demand for greater support for parents of children and adolescents with EDs, as well as the potential for PLPSGs to alleviate the stress and burden of parents, we explored the implementation of four virtual parent-led peer support groups (vPLPSGs) in Ontario, Canada. This paper reports upon the qualitative accounts of parents of children and adolescents with EDs who participated in these groups, including their experiences in and perceptions of vPLPSGs for EDs.

## Methods

Following the principles of qualitative description design [21], the present study involved individual semi-structured qualitative interviews with parents, aiming to describe their experiences and perceptions of the support groups that they attended throughout the study period.

### Setting

Each of the four virtual support groups were hosted by two parent facilitators (five parent facilitators were involved in the study in total) and included a maximum of 12 parents of children with EDs, who resided in various urban and rural regions of Ontario, Canada. Groups took place virtually using Zoom for Healthcare.

### Recruitment

**Fig. 1:**
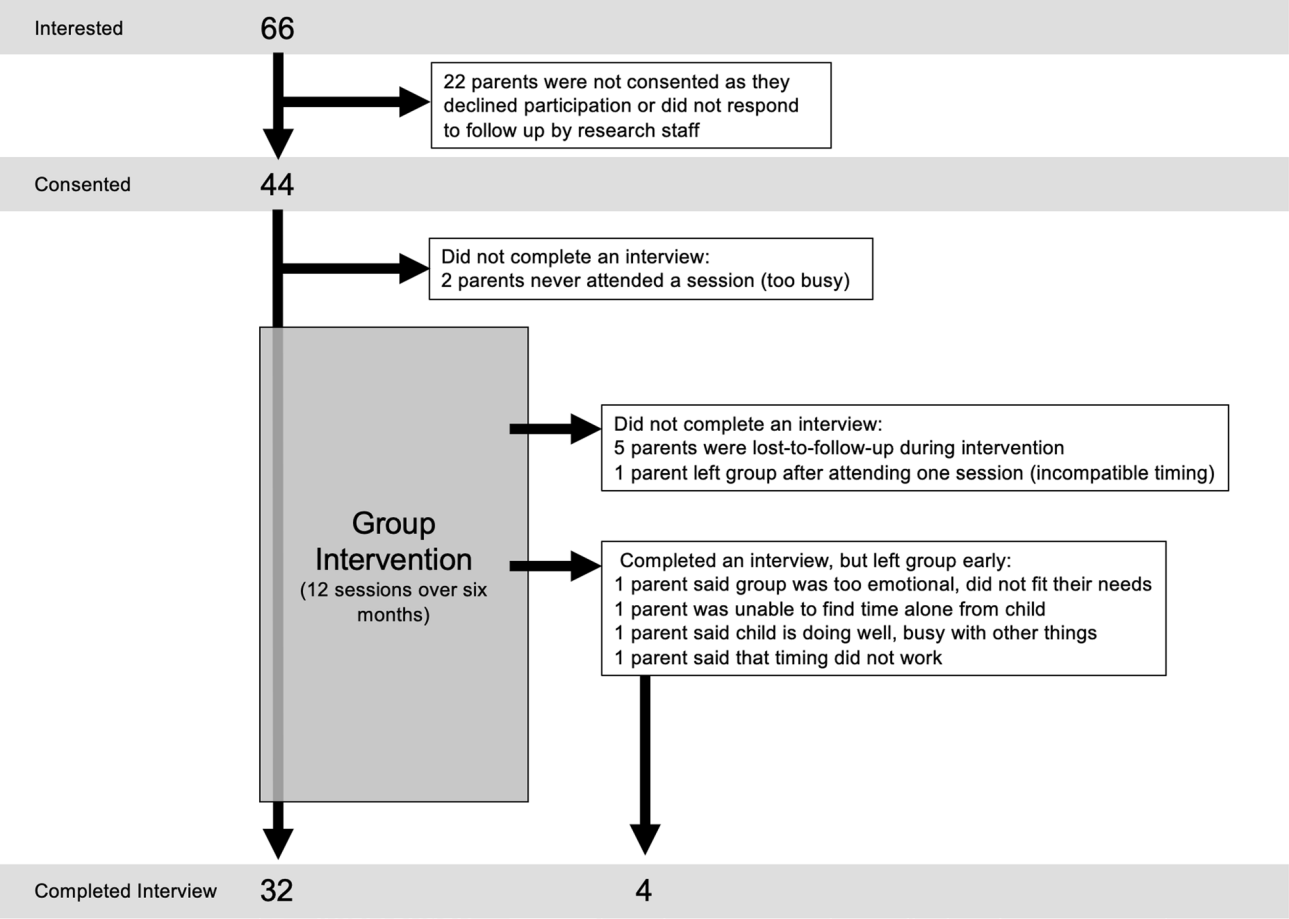
Participant flow chart

Ethics approval was received from the Hamilton Integrated Research Ethics Board in Hamilton, Ontario, Canada. Ethics approval was also received from the ethics committee at Reach Out Centre for Kids (ROCK) in Burlington, Ontario, Canada, as they were partners in conducting this project. Parents were recruited to participate in the virtual support groups via webpage posts from ED organizations and through a flyer with hospital- and community-based ED programs, including both ROCK and McMaster Children’s Hospital. Eligible participants were parents or caregivers of a child or adolescent 20 years of age or younger who was diagnosed with an ED at age 17 or younger, who lived in Ontario, Canada, were able to speak, read, and write in the English language, and had access to a working computer with internet connection. Sixty-six individuals expressed interest in the study. Of these, 44 consented to participate in the study (Fig. 1). Of the 44 participants consented, four parents withdrew voluntarily for reasons including: (a) becoming too busy to commit to the groups and study (n = 2); (b) not having privacy in their home to attend the group (e.g., did not feel comfortable to attend and participate if they felt that their child might be listening) (n = 1); or (c) feeling like they could not relate their situation to other parents (n = 1). Despite efforts by research staff, four additional parents were lost-to-follow-up; each participant was contacted by email at a minimum of three separate occasions and called at least once in an attempt to have them complete the final interview. In total, 36 parents completed the interview.

### Intervention

Parents with lived experience of having a child who recovered from an ED led the support groups and were referred to as parent facilitators. Parent facilitators learned how to conduct the groups by observing and receiving guidance from the lead parent facilitator (TL) who voluntarily conducted these groups for just over 5 years before study commencement. Parent facilitators accessed a resource repository with materials selected by the lead facilitator to provide educational content or tools for parents attending the sessions. The support group model and discussion topics (which included coping with emotions, caregiver coping styles, collaborating with the care team and school, communication skills, stages of change, externalizing the ED, suicide and self-harm) followed an evidence- and experienced-based ED caregiver education manual [22]. Groups occurred twice per month on weekday evenings and were 2 h long.

Parents were asked to attend 12 support group sessions over a 6-month period. On average participants attended 6.9 sessions, and 66.7% of participants attended 6 or more sessions (n = 24). The general structure of each session started with a check-in from each parent about their wellbeing and how their child was doing, a parent facilitator-led presentation on an education topic (e.g., how to manage your child’s ED over the holidays) that was relevant to the group’s needs with occasional guest speakers, and some time was dedicated to sharing difficulties or to seek support from others in the group. Sessions ended with a check-out where group members stated how they felt after the session and had time to ask any final questions. After the session, parent facilitators typically shared key resources via email (e.g., suggestion for websites, books, videos, etc.).

### Data collection

Semi-structured individual interviews inquiring about parental experiences in and perceptions of the groups were conducted virtually and recorded using Zoom for Healthcare. The creation of the interview guide and interviewing approach were guided by the principles of qualitative description [21] (See Additional file 1 for the interview guide). The interviews were video-recorded and transcribed verbatim.

### Data analysis

Characterized by its systematic coding and categorizing approach, content analysis was chosen to analyze the data for this study [23]. This approach provides a “straight description” of participant responses using minimal interpretation, which aligns with the qualitative description design [21, 24] and purpose of the study: to generate a description of the participants’ perceptions and experiences of the support groups. Specifically, two distinct types of content analysis—conventional and summative—were blended together to analyze the qualitative data [25]. First, conventional content analysis was applied by two separate coders (LG and MN) who inductively derived a set of initial codes—words or phrases used by participants—through multiple readings of the transcripts. All coding was completed using NVivo 10 (QSR International Pty Ltd., Version 8, 2008). In line with methodological guidelines to double-code 10–25% of the data units [26, 27], the coders double-coded 17% (six) of the transcripts. The proportion of initial conflicts was 27%; all conflicts were resolved. Together, coders collapsed and sorted codes into larger categories to guide a report reflective of the data. Using summative content analysis, the coders counted the number of participants and mentions by each participant for each perception and experience expressed in the data to provide an indication of the emphasis placed on each code or category. Representative quotes were used to support and contextualize some of the more frequently-stated perspectives and opinions in the resultant reporting of this data.

## Results

### Participants

The demographics of the 36 parents who completed the qualitative interview is reported in Table 1. Participants also provided information about their child/children who had an ED (Table 2).

**Table 1:** Demographic characteristics of study participants (n = 36)

Characteristics	Number of participants	Range (years)	Mean ± SD (years)	Percent
Geographical region of participants				
Southwestern Ontario	19			52.8
Eastern Ontario	9			25.0
Northern Ontario	8			22.2
Age		37–62	48.89 (5.98)	
Relationship to Child who has an ED				
Mother	30			83.8
Father	6			16.7
Marital Status				
Married or living as common-law	31			86.1
Divorced or separated, not remarried	4			11.1
Prefer not to answer	1			2.8
Highest Level of Education				
Completed secondary or high school	4			11.1
Non-University or College Certificate	6			16.7
Bachelor’s Degree	14			38.9
University degree/certificate above bachelor’s (e.g., Master’s, MBA, MD, DMD, PhD)	12			33.3
Yearly Gross Household Income				
$35,000-$49,999	1			2.8
$50,000-$74,999	0			0.0
$75,000-$99,999	7			19.4
$100,000-$149,999	15			41.7
$150,000-$199,999	6			16.7
$200,000 or more	7			19.4
Ethnicity				
White, Caucasian, or Canadian	33			91.7
Other	3			8.3
Parent’s History of an ED				
No	30			83.3
Yes	4			11.1
Prefer not to answer	2			5.6

*SD* standard deviation

**Table 2:** Demographic characteristics of study participants' child who has an ED (n = 36)

Characteristics	Number of participants	Range (months)	Mean ± SD (months)	Percent
Duration of ED (n = 35)		2–72	22.81 (19.26)	
Over 1 year	19			52.8
Under 1 year	16			44.4
Gender				
Female	33			91.7
Male	1			2.8
Non-binary	2			5.6
Type of ED reported by parent				
AN, restrictive behaviour	27			75.0
BN, purging behaviour	4			11.1
Combination of AN and BN	1			2.8
Combination of AN and binge eating	1			2.8
Binge-eating disorder	1			2.8
Atypical AN	1			2.8
Orthorexia nervosa	1			2.8

*SD* standard deviation, *AN* anorexia nervosa, *BN* bulimia nervosa

Of those who completed the interview, 83.8% of participants were mothers (n = 30), 91.7% of participants identified as White, Caucasian or Canadian (n = 33), 72.2% of parents had a bachelor’s degree or higher education (n = 26), and their age range was from 37 to 62 years. Parents reported on their child’s gender, with 91.7% (n = 33) of children being female, 2.8% (n = 1) being male and 5.6% (n = 2) being non-binary. Seventy-five percent of parents identified that their child had anorexia nervosa (AN), restricting type (n = 27), and 52.8% of parents reported that their child had been struggling with an ED for over 1 year (n = 19). Three participants reported that they had more than one child who was struggling with an ED—all of whom were female. The mean duration of the second child’s ED was 13.3 months (SD = 10.5), and two parents reported that the second child had AN, restricting type, while one parent was unsure of their second child’s diagnosis.

### Qualitative results

Please refer to Table 3 for a summary of the summative content analysis of the experiences and perceptions of parents regarding the vPLPSGs.

**Table 3:** Categories and subcategories emerging from semi-structured interviews with parents (n = 36 participants)

Category	Subcategory	Frequency
Overall	Group structure	13 participants, 16 references
	Group atmosphere (e.g. it’s a safe space)	11 participants, 16 references
	Group discussion topics were appropriate	8 participants, 10 references
	Group organization	5 participants, 5 references
	Group size (adequate)	1 participant, 1 reference
Parent Facilitators	Good facilitators (e.g., amazing, wonderful, great)	29 participants, 47 references
	Knowledgeable facilitators (e.g., resourceful)	22 participants, 27 references
	Supportive facilitators (e.g., caring, welcoming, empathetic)	19 participants, 24 references
	Liked and valued their lived experience	14 participants, 14 references
	Appreciated their facilitation approach	13 participants, 14 references
	Appreciated their addition of positivity	10 participants, 12 references
	Easy to communicate with outside of the sessions	7 participants, 7 references
	They fostered meaningful connection and discussion amongst group members	7 participants, 7 references
	Felt they could have managed participants better	4 participants, 6 references
	Checked in with struggling parents	4 participants, 4 references
	Co-facilitators worked well together	1 participant, 1 reference
Other Parents	Good (e.g., lovely, amazing)	18 participants, 21 references
	Supportive (e.g., respectful, compassionate)	12 participants, 17 references
	Knowledgeable	1 participant, 1 reference
	Recognized that everyone is at a different point in their journey with their child	15 participants, 16 references
	Annoyed with another parent	6 participants, 8 references
	Made connections with another parent outside of the group	4 participants, 5 references
	Inspired by other parents	2 participants, 4 references
	Groups lacked diversity	1 participant, 1 reference
Support	Felt supported by the group	31 participants, 49 references
	Helpful to know that others are going through similar things and that they are not alone	30 participants, 59 references
	Valued tips, ideas, resources, and strategies from other parents	19 participants, 39 references
	Decrease in shame/embarrassment/guilt	17 participants, 28 references
	Easier to talk to group members than other friends/family/coworkers	17 participants, 22 references
	Feelings of validation	16 participants, 26 references
	Felt comfortable speaking about their experiences	13 participants, 16 references
	Learned to prioritize their own wellbeing as a caregiver	9 participants, 14 references
	Helpful to be able to help others	9 participants, 11 references
	Helpful hearing success stories	8 participants, 9 references
	Became aware that recovery can take time, easing tension	6 participants, 6 references
	Motivated and more confident helping their child and take their child’s ED more seriously	4 participants, 6 references
Education and Resources	Valued amount and variety of resources about EDs provided	24 participants, 46 references
	Education topics were seen as being helpful in the future	12 participants, 16 references
	Post-session emails with resources helpful	10 participants, 13 references
	Appreciation for high-quality, pre-selected resources offered by facilitators	8 participants, 8 references
	Learning about types/presentations of EDs and warning signs helpful	8 participants, 8 references
	Topics were never relevant due to a difference in their child’s age/diagnosis/experience	7 participants, 10 references
	Parent wished they had access to education earlier in their child’s illness	4 participants, 7 references
	Topics became relevant during the study period	2 participants, 2 references
Less Helpful Aspects	Nothing was less helpful	6 participants, 7 references
	Overfocus on anorexia nervosa	4 participants, 5 references
	Topics repetitive	2 participants, 3 references
	Do not belong in the group because their child’s problems are not as severe	5 participants, 12 references
	Parents from rural areas unable to access resources in Southern Ontario	4 participants, 5 references
	Parents new to EDs initially scared by other children’s severity/duration of ED	3 participants, 3 references
	Do not want to feel like they are boasting when talking about successes	3 participants, 3 references
	Felt envious of other parent’s successes, comparing their child to others	3 participants, 5 references
	Emotional but helpful	6 participants, 6 references
	Sessions were too emotional at times	4 participants, 5 references
Suggestions for Change	No suggestions for change	7 participants, 7 references
	More recovery/success stories	9 participants, 13 references
	More focus on transition out of pediatric care	1 participant, 1 reference
	More focus on mental health aspects of EDs rather than physical aspects	4 participants, 7 references
	More focus on relapse prevention	1 participant, 2 references
	Separate groups based on child’s age	2 participants, 4 references
	Separate groups based on child’s ED diagnosis	4 participants, 5 references
	Separate groups based on child’s ED duration	2 participants, 4 references
	Separate groups based on geographical location	2 participants, 4 references
	Separate groups based on cultural needs (e.g., Indigenous cultural facilitator)	2 participants, 3 references
Influence of Support Group on Child who has an ED	No effect on relationship	3 participants, 4 references
	Relationship improved	21 participants, 31 references
	Used skills they learned in group with their child (e.g., changing language)	28 participants, 58 references
	Helped parent become more patient with child	8 participants, 9 references
	Eased parents’ expectations related to the ED (e.g., slips/mishaps are normal)	8 participants, 9 references
	Helped parents externalize the illness	6 participants, 7 references
	No effect on ED symptoms	8 participants, 8 references
	Improved symptoms	2 participants, 2 references
Influence of Support Group on Siblings	No impact	11 participants, 11 references
	More aware of how siblings can be impacted and how to support them	14 participants, 23 references
	Check siblings for warning signs of EDs/use preventative measures	6 participants, 8 references
	Prioritize spending time alone with siblings	5 participants, 6 references
	Siblings happy that parent was getting support	3 participants, 4 references
Availability and Accessibility	Groups should be advertised and supported by hospitals and community organizations	26 participants, 52 references
	More of these groups should be offered	11 participants, 12 references
	Groups should be peer led	4 participants, 4 references
	Parent facilitators should be fairly compensated	9 participants, 10 references
	Surprised that these groups are not already commonplace	1 participant, 1 reference

#### Overall experience

Participants were asked about their overall experience in the group. Numerous parents described the group as a welcoming environment, indicating that overall, it was generally helpful, supportive, and valuable:

Honestly, it’s felt like a little bit of a lifeline for me… I can’t imagine if I didn’t have the group and trying to manage this alone. (Parent 4).

Some indicated that they had no other forms of support to help them with their child’s disorder:

To be honest, this was really our only support that we had during this period of time. Didn’t have any other counsellors. [My daughter] talked to a counsellor a couple of times during the period, and then obviously doctor’s appointments with our doctor here. But this was our main support. (Parent 35).

Many parents hoped that the groups would continue in the future, and expressed gratitude for the existence of the group. One parent stated:

It came into my life right when I needed it and it’s been… it’s hit all those points that you need as a parent – education, compassion, validation, all that kind of stuff. So I think it’s been really great. (Parent 24).

Participants reported that they liked the group size as well as structure and organization, including pre-session emails that contained a plan for the session and post-session emails with resources discussed. Individuals also appreciated that discussion topics were chosen based on the needs of the group. The comfortable, nonjudgmental, supportive atmosphere was notable, as one participant remarked:

… it was the first time I could speak really completely candidly and it was a safe environment. (Parent 9).

#### Parent facilitators

Group members appreciated that the parent facilitators had lived experience of having a child with an ED and had numerous positive impressions of the facilitators. Thirteen participants also indicated that facilitators managed the groups well. For example, reports were made that the facilitators skillfully fostered meaningful discussion and connection amongst group members, giving each parent an equal chance to speak and share, while also keeping conversations focused; however, one participant stated that the facilitators could have been stricter in preventing others from taking up time speaking about unrelated topics. Ten participants were grateful that the facilitators tried to add positivity to groups, such as sharing success stories when appropriate. It was also reported that the facilitators were easy to communicate with outside of the group when necessary, for example, providing extra information and resources as needed and following up with struggling parents:

After the first session, or during the first session, I was upset at one point, and she called me after the session to make sure that I was okay. (Parent 1).

#### Other parents

When asked to describe their experience with other parents in the group, it was noted that the majority of participants were mothers of teenage girls. Parents noted that their peers had older and younger children, children with different severities of EDs, children diagnosed recently or years ago, and different personalities. Parents were aware of the differences between their own experiences with their children and others’ experiences, explaining how everyone was at a different point in their journey:

Just a broad spectrum of different parents who, the main thing that they wanted was to help their children and be strong for their children, and in some way learn ways to be better parents. (Parent 5).

Most participants appreciated their differences in experience and claimed to have learned a lot by listening to others. One participant noted that the groups lacked diversity:

I did find it was overwhelmingly white, so not terribly diverse in terms of socioeconomics or ethnicity…And I know that anorexia tends to attack certain [laughs] demographics, but I don’t know if that’s something you’re assessing or evaluating. But I mean if I was a person of colour or something like that, I would find that to be a barrier. (Parent 24).

Similar to their reports of the parent facilitators, parents generally had positive things to say about one another and felt inspired by others in their group, where all members appreciated the open sharing and vulnerability. Many parents became invested in one another’s stories and journeys, with four parents making connections that translated outside of the group. Aside from mostly positive comments, a few participants noted becoming annoyed with one or two other parents due to different communication styles and personalities.

#### Support

Thirty-one participants commented on how the group made them feel well-supported, and that it was helpful to know that they were not alone and others were managing similar struggles. Parents also appreciated having a space to vent, stating:

I did feel like when I finished, kind of that same relief as when you had that opportunity to just speak to a counsellor. (Parent 17).

Parents reported feeling free to speak about their experiences and grateful for support and encouragement from other participants. Nine participants also learned to prioritize their well-being as a caregiver. Several parents reported feeling validated when they were reassured by others that they were doing an adequate job caring for their child. Notably, a decrease in shame, embarrassment, and guilt were reported by seventeen parents. For example, one parent stated that they felt less blame in relation to their child’s ED and their management of it thus far as a result of the group:

You could tell yourself a hundred times, ‘It is not your fault,’ but you always feel like you could have done something better, you could have done… what did you miss, or… you know? But being in this group, you didn’t feel that way. You felt normal. (Parent 6).

Participants also expressed that the group motivated them to take their child’s ED seriously, and to become aware of different phases of the illness, accepting that recovery can take a while. Hearing success stories from the parent facilitators and other group parents was particularly helpful in promoting hope and optimism. Other aspects of the group that reportedly fostered feelings of support for nineteen participants included the sharing of tips, ideas, strategies, and resources that they received from fellow group members. For example, one parent stated:

… I found it really helpful to talk to the other parents with a lot more experience, to get some guidance from them about what’s really going on here. And 99% of the time, they were exactly right about what was going on. So that was really good to know and helpful to have. (Parent 24).

Seventeen participants also discussed the value of having support specifically from parents with children who have an ED, which is distinctive from the well-intentioned support received from others without this lived experience, such as friends, family, and colleagues:

I can talk to my family, I can talk with friends. It’s all fine. But nobody gets it. Nobody really gets it. But when you get in that group, everybody gets you. You’re going to hear stories that you were planning to tell from other people before you do your own story, because a lot of the stories are the same. And just for that reason, to be able to share with people that gets it to me is huge. (Parent 13).

Beyond receiving support from the group for themselves, many participants enjoyed being able to help others due to their breadth of experiences, or by the end of the group felt more equipped to help others:

I needed a lot of help and support initially, but now as it’s transitioned, I feel like I’m now able to offer help and support, and that’s really a good feeling. (Parent 9).

#### Education and resources

Another helpful component of the groups that most parents commented on was related to the resources, education, and information they received about EDs. More specifically, twenty-four parents found the teachings and resources related to ED types and warning signs of disordered behaviours to be the most helpful aspects of the groups. Ten parents found the post-session emails attached with resources that were used during the session to be particularly helpful. These emails enabled parents to read through the resources at their own pace, or refer back to topics when needed. In particular, eight parents mentioned feeling grateful to have access to the high-quality resources provided by parent facilitators:

Definitely all the resources that they found for you so you didn’t have to go searching for yourself, plus their own experience, their own knowledge offhand, right? You can ask them a question, and they can give you a really good thought-out answer right there. So, you don’t have to go look for yourself. Everything is just handed right to you, so it was great. (Parent 33).

A portion of the topics discussed were not directly relevant to all participants due to varied diagnoses, stages of recovery, and other factors. Despite this, twelve parents commented on the value of the information they received, recognizing that it might be helpful in the future:

And I was never really in a session where I was ever thinking, ‘Oh, this doesn’t resonate with me’ or… I mean there’s definitely topics where probably my daughter hadn’t really experienced that, but I was kind of keen to learn just because I know that this disease is kind of a long-haul thing. And so I always sort of found I was intently listening because, even though my daughter wasn’t going through this now, I thought, ‘Well, she might in the future.’ (Parent 25).

For some, it did become helpful later in the group:

Yeah, and we never dealt with purging until very recently. I’ve learned that she’s been trying to purge. She hasn’t been successful yet, but six months ago that topic wouldn’t have been relevant to me and now it is. (Parent 16).

Some parents who did not find the topics particularly helpful mentioned that this was because they would have liked to have had this information earlier in their child’s illness, but still appreciated the reminder. Seven parents mentioned that they found some content to be irrelevant:

The only thing is sometimes there’d be a talk. There was one on binge eating or bulimia, and it just didn’t pertain to me. It was still interesting, but it didn’t relate. (Parent 9).

#### Less helpful aspects

Although some parents only commented on the helpful nature of the support group and had no negative aspects to report, others offered their insights on less helpful aspects. For example, four such parents noted that the group discussion of certain topics was not helpful, such as a greater focus on AN and weight restoration, which was not always applicable for parents with children living with a different ED (e.g., if their child had bulimia nervosa). Two participants perceived some topics as repetitive. A few participants felt that their child’s problems were not as severe as others, that perhaps they did not “deserve” to be there, or that they did not want to come across as boastful when talking about their successes with their child:

I felt like sometimes other people in the group felt like I shouldn’t be there because we didn’t have the same kind of heavy diagnosis. (Parent 31).

In contrast, some parents reported feeling envious of other parents’ successes with their children, comparing their own child to others. Additionally, parents from rural areas of Ontario found it difficult to listen to discussions about resources in urban areas, given their own lack of access to support.

Some participants stated that the initial sessions felt more emotional for them than the sessions that came later, especially when hearing stories from other parents. Additionally, a group of parents found that the difficult emotions they experienced detracted from their positive experiences, with some reporting information overload or feeling overwhelmed; this contributed to one parent leaving the group:

… it was emotional for me, it was just one thing that I needed to release in order to deal with the other things more effectively. So it had nothing to do necessarily with the group. It was definitely just not a time for me to participate in something like that. (Parent 26).

#### Suggestions for change

Several participants made recommendations with regards to session content. For instance, some parents reported that they wanted to hear more recovery and success stories, or to hear directly from recovered young adults:

I think maybe what I need more… some more success stories. That would be it, because it’s hard when everybody’s right in the trenches of it, and a lot of people, there isn’t a lot of light. And I think it would be good to even, to just be reminded that people do come out of this, and they come out of this better or stronger, but it’s not necessarily… a forever thing. (Parent 19).

Furthermore, several parents noted that having a greater focus on how to transition their child out of pediatric care, the mental health implications of EDs in addition to physical health issues, and relapse prevention after hospitalization would have been useful. Ten participants suggested that future groups should be organized based on the age of the child with the ED (e.g., 10–13 vs. 15–17), the ED duration (e.g., less than 1 year vs. 3+ years), type of ED, and geographical area. One parent noted that it would be helpful to have a culturally-specific facilitator to support minority and marginalized groups, such as Indigenous peoples:

So having a different… a support group that might have an elder or might talk about intergenerational trauma or residential school syndrome I think would be a pretty good set-up. (Parent 35).

#### Influence of support group on child with an ED

Many participants found that the group improved their relationship with their child, stating that it improved their communication, helped them fight less, enabled them to prioritize time which was not spent talking about the ED, and allowed parents to externalize the illness (i.e., see their child as separate from their ED). Parents also reported modeling behaviour that they wanted their child to engage in, using skills they learned in group with their child, and being mindful of language they use around their child:

All of that makes you much more aware of the things that are influencing your child, even from harmless family conversations you never would have thought twice about. Now like ‘You can’t say that’ or ‘We don’t use those words’ or ‘We don’t talk about food in that way’ (Parent 4).

Participants reported that the group helped them become a better listener, more patient, less frustrated when communicating with their child who has an ED, and eased their anxiety and expectations related to the ED. One parent expanded on this, saying:

I really wanted her to see that her dad and I were trying to do everything we could to help her, especially because there were times that neither one of us handled things very well. You know, we were new to it. We were scared. We were overwhelmed…So, I think it helped our relationship and helped her understand that we were not just making things up as we went (Parent 12).

When asked whether the group influenced their child’s ED symptoms, many felt that the group did not impact their child’s ED directly. However, this was not an expectation they held:

There’s nothing that I can say or do to my child that will make them think any differently of their situation…The benefits that have come from this support group I think have been just felt by myself personally and how I am able to manage the expectations of being a parent of a child with all of these needs (Parent 3).

#### Influence of support group on siblings

Fourteen parents reported that the group led them to be more aware of the impact that their child who has an ED can have on their siblings and engaged in ways to manage this. For example, as a result of the skills learned in the group, some parents now monitor their other children for warning signs of an ED and have started to use preventative measures, while others have become aware of the importance of spending alone time with the siblings. Three parents reported that their other children were happy that they were getting support:

… they’re at an age now where they’re affected by the illness and they also totally want me to get support and resources. So they’re happy about the group in the sense that they know it’s going to be helpful overall for the family. (Parent 28).

#### Availability and accessibility

Twenty-six participants unanimously agreed that these support groups should be advertised and delivered by hospital programs and community health organizations. For instance, almost three-quarters reported that more peer-led groups should be offered and that they are desperately needed for parents of children who have EDs:

… you need to have a group like this where you’re welcomed and you’re comfortable, you’re accepted, you share stories, you learn, and you feel [em]powered. It should be provided to everybody. It should be out there, and it is sad not to see it. (Parent 6).

One parent stressed the need for support groups such as these, as they believed that general hospitals—lacking ED-specific units—do not give adequate information for parents after a child with an ED has been medically stabilized:

Because as a parent, I felt so lost. When you leave the hospital with a newborn baby, everybody’s got advice. Everybody’s got pamphlets … The nurse is calling you and coming to visit you a week later. There’s all sorts of information out there. When you have a teenager who almost died from an eating disorder, they’re like, ‘Okay, goodbye, good luck,’ right? (Parent 12).

Many parents indicated strong support for hospital and community organizations to have these groups, seeing them as an important component of the continuum of ED care:

If they can offer more of them and the government is willing to do that, then that would be great because if it only can happen because it’s a study, then that’s too bad. I think they’re valuable. I think people need to know that it’s a valuable experience if they’re having struggles and don’t have any connections, yeah (Parent 17).

Another parent shared:

… I think it should be one of the pillars of the management of people with eating disorders. (Parent 23).

In addition to supporting the widespread availability of this group, parents also identified that they believed the group should continue to be run by a peer facilitator and not have external involvement from healthcare professionals, as it could impact group dynamics:

I think what happens with parent support groups, you tend to probably have a less hierarchical dynamic. So people are sharing and… When you have a professional, it’s [a] more hierarchical dynamic. (Parent 27).

Participants acknowledged that healthcare professionals are helpful in supporting their child, but that they are not expected to and not able to provide peer support for the parents:

I just think it gives parents an opportunity to share and learn in a really meaningful way from people who have been there, done that, or are going through it at the same time, which you don’t get that from professionals. Even [eating disorder clinician], I love her to bits and she’s helpful and wonderful, but she doesn’t help me navigate my experience with eating disorders. Right? (Parent 16).

## Discussion

This is the only study to qualitatively explore parents’ views and experiences after participating in vPLPSGs for parents of children and adolescents with EDs, with the exception of a recent study examining peer led groups for parents of children with EDs and comorbid ASD [14]. Parents generally described having remarkably positive experiences related to organizational features of the group, the parent facilitators and other attendees, and the education and resources provided. Most commonly, parents reported that the groups fostered a high degree of much needed support reporting that they felt less alone, they connected with others who truly understood their situation, and they experienced a decrease in guilt and shame of having a child who developed an ED under their care.

Parents especially valued learning tips and strategies from other group members and receiving high-quality and practical resources from their facilitators. As the COVID-19 pandemic and its associated restrictions resulted in reduced access to ED services and social supports [28], it was especially important that parents received guidance and support to help themselves and their child. These findings revealed that it can be overwhelming for parents, including both parents who are new to the illness and those well-versed in EDs, to independently navigate and find appropriate resources. As the pandemic has also increased the responsibilities of those caring for an individual with an ED [6, 28], parents may lack the time necessary to sift through resources to identify the most valuable and evidence-based options.

Many participants indicated that the group improved their relationship with their child who has an ED and made them more aware of the impact on their other children. This is a noteworthy finding, given that past research suggests that EDs place a significant strain on family relationships and dynamics [29–31]. Individuals who reflected on their ED recovery stated that family members that are non-judgemental and supportive create relationships that are particularly influential in their recovery [32].

A few participants noted the lack of diversity in participants’ socioeconomic status (SES) and ethnicity. This is especially relevant, as despite historical stereotypes, EDs are not restricted to affecting individuals of high SES, warranting the need for more research on support for marginalized groups [33]. It is also widely known that EDs affect individuals of all racial and ethnic backgrounds, but have been underrepresented in the literature for several decades [34]. Future research should focus on the experiences of individuals from ethnic or racial minority groups to determine their needs as caregivers. Our results also highlighted the importance of adapting support groups to meet the needs of caregivers who belong to different cultures, including Indigenous individuals. It has been noted that when conducting peer support groups with parents of children with disabilities, cultural training and competency of facilitators is important to meet these individuals’ needs [35]. For this, we believe that future studies should include cultural training for parent facilitators, and prioritize recruiting individuals from a variety of cultural identities to better serve diverse participants.

The majority of parents believed that these vPLPSGs should be widely available, indicating that they are a pivotal part of the continuum of care in pediatric EDs. Currently, there is a lack of support for parents despite a stated need for more peer support, as it assists them in being able to manage their child’s ED [36]. Parents are also burdened by long waiting lists at various stages of pediatric ED care and face challenges in accessing ED services, harbouring emotions of guilt and self-blame [37]. The widespread implementation of groups such as these could provide support and education to parents while they are on waitlists, and assist in managing their negative emotions so they are capable of focusing on supporting their child’s recovery.

One dominant suggestion for improvement made by participants included having more success and recovery stories incorporated in group content. Caregivers of individuals with EDs experience higher levels of negative affect, including depression, which increases their caregiver burden [38]. Focusing on more success and recovery stories may instill and maintain hope in caregivers and help them to maintain more positive mental health to be able to support their loved one. Parents also recommended that future groups could be organized according to their child’s age, ED duration, ED diagnosis, and geographical location. For instance, dividing groups based on ED duration may reduce the shock and stress parents that are newer to EDs feel. Groups could be split with one group comprised of parents who have a child that has had an ED for 2 years or less, and another where their child has had an ED for more than 2 years. Organization based on diagnosis may enable parents to access more resources that pertain to their child’s particular ED and be around others who have more similar experiences. For instance, a group based on AN would include topics related to weight restoration, while a group based on bulimia nervosa would focus on supporting the reduction of purging behaviours. Further division based on geographical area may enable parents to be exposed to local resources and programs that can support them. These suggestions can be incorporated in the future and merit further study to determine any differences in parent outcomes.

Studies of PLPSGs in other contexts have found similar positive results. A study of PLPSGs for parents/carers of children with attention deficit hyperactivity disorder (ADHD) found that after attending six sessions, group members reported feeling less isolated and alone in their struggles, and more confident and connected to others as a result of being in a supportive space with others who have similar shared experiences [39]. Qualitative analyses in a systematic review of one-to-one and group peer support for parents of children with chronic, disabling health conditions identified four themes related to the benefits of: belonging and support from meeting with others with similar lived experiences, learning from other parents’ expertise and experiences, developing skills and feeling more self-empowered and confident, and being able to offer others support in boosting self-efficacy and self-worth [40]—topics which were echoed by parents in the present study. Another study that evaluated peer-to-peer support for parents of at-risk youth who have behavioural and emotional difficulties found increases in parents’ perceived social support and concrete support [41]. Our findings contribute to the literature that promotes parent-led peer support for parents of children with complex needs.

Notable strengths were apparent in our study. Unlike many qualitative studies, a portion of our interviews included some individuals who withdrew from the group. This offered a different perspective compared to study completers. Our sample was also geographically diverse as we recruited parents across the province, including urban and rural settings, which allowed for a truer representation of our population. The virtual nature of the groups enabled us to recruit a geographically diverse sample, removed potential transportation barriers, and enabled participants to take part in the groups despite changing COVID-19 pandemic restrictions. Finally, our sample size was large for a qualitative study [42], which allowed for a wider variety of perspectives to be captured in the data.

Limitations were also present in our study. Despite some geographical diversity, we had unequal representation of parents, recruiting more participants from southeastern and southwestern Ontario compared to northern Ontario. We also lacked a diverse sample in terms of ethnicity, with the majority of participants self-identifying as White or Caucasian, limiting perspectives received. We were unable to interview every participant due to loss to follow-up, which also may have included different perspectives and suggestions. Although participants were asked to attend twelve groups in total, participants attended six on average, which may have limited the extent of their feedback due to reduced attendance and experience in group. Future studies may implement a shortened duration of groups as parents might have found attending twelve groups too onerous. Additionally, the virtual nature of the groups meant that individuals had to have a computer or other device with internet connection in order to participate, which posed a barrier to participation amongst those without such access.

## Conclusions

Our findings suggest that vPLPSGs are well-received by parents of children and adolescents with EDs. Participants approved of the group organization, valued the kindness and knowledge that the facilitators displayed, and found the support, education, and resources received from the groups helpful. An overfocus on AN, emotional heaviness, and social comparisons were difficult for some parents. Some of these issues can be ameliorated by implementing suggestions made by the participants such as including more recovery and success stories, and organizing groups according to the child’s age, ED diagnosis, duration of illness, and participants’ geographical location. Many parents reported that attending the groups improved their relationship with their child who has an ED, as they used skills learned in session when interacting with them, and that siblings also benefitted as parents were more aware of how they are impacted and how they can be supported. Participants unanimously agreed that vPLPSGs should be widely available, maintaining the peer support model with experienced and trained parent facilitators leading the groups.

## Supplementary Information

**Additional file 1.** Post-Intervention Semi-Structured Interview Guide.

## Data Availability

De-identified data from this study are not available in a public archive. There is no analytic code associated with this study. Material used to conduct the study are not publicly available.

## Abbreviations

ED: Eating disorder
PLPSGs: Parent-led peer support groups
SES: Socioeconomic status
vPLPSGs: Virtual parent-led peer support groups
