# Supplementary material for: “I’m not alone”: a qualitative report of experiences among parents of children with eating disorders attending virtual parent-led peer support groups
Source: J Eat Disord. 2022 Dec 15;10:195. doi: 10.1186/s40337-022-00719-2 (PMC9754305; doi:10.1186/s40337-022-00719-2)
Supplement: Supplementary file 1 — Additional file 1. Post-Intervention Semi-Structured Interview Guide. [file 40337_2022_719_MOESM1_ESM.docx]

**Additional File 1**

**Post-Intervention Semi-Structured Interview Guide**

Introduction

*Thank you for participating in our study and in the parent support group. It is estimated that this interview will take a maximum of 60 minutes. During this final interview, I hope to gather an understanding of your experience in the group. Please know that you are not required to answer any questions that you would like not to answer.*

Description of Experience in the Group

- *What led you to join or reach out about this group—for parents of children who have an eating disorder?*
- *How would you describe the parent support group itself?*
  - Prompts: *What was the format of your particular group? Who was present at the group sessions? What topics were discussed in the group?*
- *How did you feel about your parent facilitator, (name)?*

*How did you feel about the other parents?*

Acceptability

- *What were the most helpful parts during your time in the group, if anything?*
  - Prompts: *What new things did you learn, if anything? Did any aspects of the group stick out for you? Did you make connections with parents? What was your experience hearing new perspectives?*
- *What were the least helpful parts during your time in the group, if anything?*
  - Prompts*: For instance, were sessions emotional or were you unable to relate to other parents/topics being discussed?*
- (If applicable) *Would you be able to elaborate upon why you left the group early?*
- *What would you change about the group, if anything?*
  - Prompts: *How could it be improved for other parents like you?*

Parent Outcomes

- Isolation
  - *How did your time in the group influence the feelings of isolation you may have experienced prior to participating in the group?*
- Burden
  - *Similarly, how did your time in the group influence the feelings of burden you may have experienced prior to participating in the group?*
- Self-efficacy
  - *How did the group influence your confidence in your ability to help your child(ren)?*
- Hope
  - *How did the group influence your feelings of hope compared to before you participated in the group?*
- Skills
  - *What skills, if any, did you acquire throughout your time in the group?*
  - *Did you learn anything new? If so, what did you learn?*
- Effect on Children
  - *How do you feel that your child’s eating disorder influenced your experience of the group?*
    - Prompts: *When they were not doing as well? When they were doing better?*
  - *How do you feel your participation in this group influenced your relationship with your child with the eating disorder? Why or how come?*
  - *How do you feel your participation in this group influenced your child with the eating disorder in general? Why or how come?*
    - Prompts: *How do you think your participation in this group influenced your child’s eating disorder symptoms or experience?*
  - *How do you feel your participation in this group influenced your other children? Why or how come?*

Cost

- Time spent: *If you were to estimate the time you spent in the group, how many hours would you say you contributed?*
- Sacrifices: *Did you have to make any sacrifices to attend the group?*
- *Did you have to navigate other childcare and/or other responsibilities in order to attend?*
  - (If applicable) *How did you navigate this?*

COVID-19

- *Do you think that the COVID-19 pandemic played a role in you reaching out to this group? Why or why not?*
- *Do you think that the COVID-19 pandemic played a role in your attendance to this group? Why or why not?*

Format

- *What are your thoughts about the virtual format of the group?*
  - Prompts: *What did you like about the group being run online? What would you change for next time? How do you think this would compare to an in-person group?*
- *What are your thoughts about…*
  - *the frequency (bi-monthly) of the sessions?*
  - *the length (2 hours per meeting) of the sessions?*
  - *the set dates (e.g., Wednesdays at 7 PM) of the sessions?*

Final Thoughts

- *Overall, how would you describe your time in the group?*
- (If applicable*) If given the opportunity, would you continue attending this group?*
- *Do you think that these parent-led support groups should be advertised by other organizations (e.g., hospital programs, community health organizations)? Why or why not?*
- *Do you think that these parent-led support groups should be offered, led, or delivered by other organizations (e.g., hospital programs, community health organizations)? Why or why not?*
- *Would you recommend this group to another parent? Why or why not?*
- *Do you have any final thoughts or words you would like to share about your experience in the study and/or the group?*

*Thank you for completing this interview.*
